# Mitochondrial dysfunction in PRRSV-2-infected macrophages

**DOI:** 10.3389/fimmu.2025.1670488

**Published:** 2025-10-15

**Authors:** Thien-Phong Vu Manh, Alba Frias-De-Diego, Abigail Williams, John Byrne, Chaitawat Sirisereewan, Julie Hicks, Hsiao-Ching Liu, Elisa Crisci

**Affiliations:** ^1^ Aix Marseille Univ, CNRS, INSERM, CIML, Marseille, France; ^2^ College of Veterinary Medicine, Department of Population Health and Pathobiology, North Carolina State University, Raleigh, NC, United States; ^3^ College of Agriculture and Life Sciences, Department of Animal Science, North Carolina State University, Raleigh, NC, United States

**Keywords:** PRRSV-2, pig, macrophages, mitochondrial dysfunction, seahorse technology, transcriptomics, NanoString

## Abstract

**Introduction:**

Porcine reproductive and respiratory syndrome virus (PRRSV) is one of the most economically devastating viruses for the global swine industry. PRRSV has a known tropism for lung macrophages, where it causes impaired immune responses. This study evaluated the metabolic and immune profiles of primary porcine alveolar macrophages (PAMs) and pulmonary intravascular macrophages (PIMs) infected with different strains of PRRSV-2 isolated from North Carolina (NC) pig herds (NC134, NC18-9–7 referred to as NC174, and NC20–1 referred to as NC144), and VR2232, a PRRSV-2 prototype strain.

**Materials and methods:**

Primary enriched mononuclear phagocytes were infected *in vitro* with NC134 and NC174, sorted, and processed. The total RNA was used for a transcriptomic approach; additionally, gene expression was further validated using RT-qPCR and NanoString technology. Complementary functional assays with additional NC strains were used to further investigate the mitochondrial and metabolic dysfunction, as well as the oxidative stress induced by PRRSV-2 infection.

**Results:**

PAMs infected with both NC PRRSV-2 strains NC174 and NC134 showed similar transcriptomic profiles during the early stage of infection, with downregulation of genes involved in the oxidative phosphorylation and electron transport chain pathways. PIMs infected with both NC174 and NC134 strains showed limited alteration in the transcriptomic profiles compared to uninfected cells. Genetic reprogramming matched the PRRSV-2-induced mitochondrial impairment observed in functional assays performed using Seahorse technology. Mitochondrial respiration displayed slightly different profiles between PIMs and PAMs infected with the different PRRSV-2 strains, with PAMs showing a more substantial decrease in mitochondrial fitness compared to control cells. When reactive oxygen species (ROS) and nitric oxide (NO) production were evaluated, no differences were observed between PRRSV-2-infected PAMs and PIMs and control cells.

**Conclusion:**

These results provide valuable insights into the pathogenetic mechanism of different NC PRRSV-2 strains by focusing on the alteration in mitochondrial function in lung macrophages during early infection and highlighting differences in lung macrophage responses to distinct PRRSV-2 strains.

## 1 Introduction

Porcine reproductive and respiratory syndrome (PRRS) is one of the most deleterious diseases for swine producers. A recent analysis has shown that PRRS caused an estimated loss of $1.2 billion per year in the US industry from 2016 to 2020 (1). PRRS virus (PRRSV) is an RNA virus belonging to the *Arteriviridae* family and is classified into two species, *Betaarterivirus europensis* (PRRSV-1) and *Betaarterivirus americense* (PRRSV-2), with PRRSV-2 being predominant in North America (2, 3). Within these species, PRRSV-2 exhibits considerable genetic diversity, with strains varying in virulence and distribution, and further divided into several lineages based on ORF5 sequences (2, 4, 5).

A key feature of PRRSV pathogenesis is its tropism for cells of the monocyte lineage, particularly macrophages. The swine lung contains two primary resident macrophage populations: porcine alveolar macrophages (PAMs), which are located in the alveoli, and pulmonary intravascular macrophages (PIMs), which are embedded within the endothelial wall of lung capillaries. While these populations share similar phenotypes and genetic signatures, their distinct anatomical niches confer specialized functions (6–8). PAMs serve as the first line of defense against airborne pathogens, while PIMs are primarily involved in the clearance of blood-borne particles and can contribute to acute lung inflammation by secreting pro-inflammatory cytokines in response to systemic pathogens (8–11).

PRRSV infection of PAMs and PIMs can lead to dysfunction—characterized by impaired immune responses and altered host gene expression—which significantly contributes to PRRSV-associated respiratory disease (12–15). Previous studies have shown that while PRRSV replicates to similar levels in both PAMs and PIMs, infected PIMs tend to induce a more potent inflammatory response that can lead to endothelial barrier dysfunction (6, 12–15).

Mitochondria, essential organelles within cells, play a crucial role in bioenergetic metabolism, redox homeostasis, and apoptotic regulation, serving as a signaling platform for innate immunity. They are also an important source of reactive oxygen species (ROS). Viruses can exploit mitochondrial functions to support their infection cycle and induce oxidative stress, thereby promoting viral replication (16, 17). Oxidative stress, characterized by an imbalance between the production of oxidants and antioxidant defenses, disrupts normal cellular function and can damage biological systems (18). This imbalance has been implicated in the pathogenesis of numerous diseases, including viral infections such as HIV, influenza virus, hepatitis B and C, enterovirus A71, SARS-CoV-2, and ZIKV (16, 17, 19). Tissue damage produced by oxidative stress has been reported for influenza (20, 21), hantavirus (22), and herpes simplex virus (23) and has also been associated with viral pneumonitis (24), as well as PRRSV-mediated pathology in the lung of infected animals (25).

PRRSV infection has been shown to induce significant mitochondrial dysfunction (26–28). This dysfunction contributes to oxidative stress, characterized by elevated levels of ROS, reduced mitochondrial transmembrane potential, and morphological alterations in mitochondria, contributing to apoptosis or necrosis of infected and neighboring cells (25–28).

Virus-induced activation of phagocytes is associated with oxidative stress not only because ROS are released but also because activated phagocytes may also release pro-inflammatory mediators such as TNF-α, IL-1, and IL-6 (19, 29). Previous transcriptomic studies of PRRSV infection have described the modulation of several cellular functions and pathways. These can be broadly categorized as follows: i) immune suppression, through silencing of antiviral functions in both innate and adaptive immune cells (30–36); ii) dysregulation of cytokine production, TNF-α, IL-1, IL-6, and IL-10, including type I interferons (37, 38); and iii) impairment of macrophage functions and metabolism often through mitochondria-mediated pathways (12, 26, 27, 39). Recent findings have also indicated that the PRRSV envelope glycoprotein 5 (GP5) can manipulate endoplasmic reticulum–mitochondria contact, leading to excessive mitochondrial Ca^2+^ uptake, mitochondrial dysfunction, and mitochondrial ROS production, which subsequently activates autophagy to modulate innate immunity and support viral replication (28).

This study analyzed lung macrophage responses upon infection with different PRRSV-2 strains isolated from swine herds in North Carolina. We focused on the characterization of the transcriptomic signatures and metabolic profiles of *in vitro* PRRSV-2-infected primary macrophages with a specific emphasis on oxidative stress and mitochondrial function. Functional validations for the impaired pathways were performed using Agilent Seahorse technology and ROS and NO assays, with complementary NanoString and RT-qPCR gene expression approaches. This study has developed a new Seahorse approach to evaluate mitochondrial dysfunction during PRRSV-2 infection to increase the understanding of the molecular pathogenesis of the virus and its involvement with oxidative phosphorylation and cellular metabolism in alveolar and pulmonary intravascular macrophages.

## 2 Methods

### 2.1 Cells

PAMs and parenchymal PIMs were isolated from the lungs of pigs negative for *Mycoplasma* spp., influenza A virus, and PRRSV, which were 8–12 weeks of age with mixed sex as previously described (40–42).

Briefly, PAMs were obtained through bronchoalveolar lavage (BAL) using Phosphate Buffered Saline (PBS) supplemented with 100 IU/mL penicillin (GenClone, San Diego, CA, USA), 100 mg/mL streptomycin (GenClone, San Diego, CA, USA), and 2 mM Ethylenediaminetetraacetic Acid (EDTA) (Thermo Fisher, Waltham, MA, USA). Then, cells were centrifuged and resuspended in RPMI-1640 media supplemented with 10% Fetal Bovine Serum (FBS) (Biowest, Bradenton, FL, USA), 100 IU/mL penicillin (GenClone), 100 mg/mL streptomycin (GenClone), and 2 mM glutamine (Gibco, Waltham, MA, USA), referred to as complete RPMI. Cells were stored in freezing media (FBS + 10% Dimethyl Sulfoxide (DMSO)) at −80 °C for functional assays or used fresh for RNA-seq.

After BAL collection, PIMs were obtained by digesting the lung parenchyma (PAR) with a combination of DNase I (0.1 mg/mL, Roche, Indianapolis, IN, USA), collagenase D (2 mg/mL, Roche), and Dispase (1 mg/mL, Invitrogen, Carlsbad, CA, USA). Tissues were then crushed and filtered using 100-µm cell strainers prior to the treatment with red blood cell lysis buffer following the manufacturer’s instructions (ChemCruz, Santa Cruz, Dallas, TX, USA). Finally, cells were stored as PAR cells at −80 °C or used fresh for RNA-seq. Prior to each experiment, PAR mononuclear phagocytes (MNPs) were enriched using a 1.065 density iodixanol gradient (OptiPrep, Stemcell, Vancouver, BC, Canada) as previously described (40–42).

**Figure 1:**
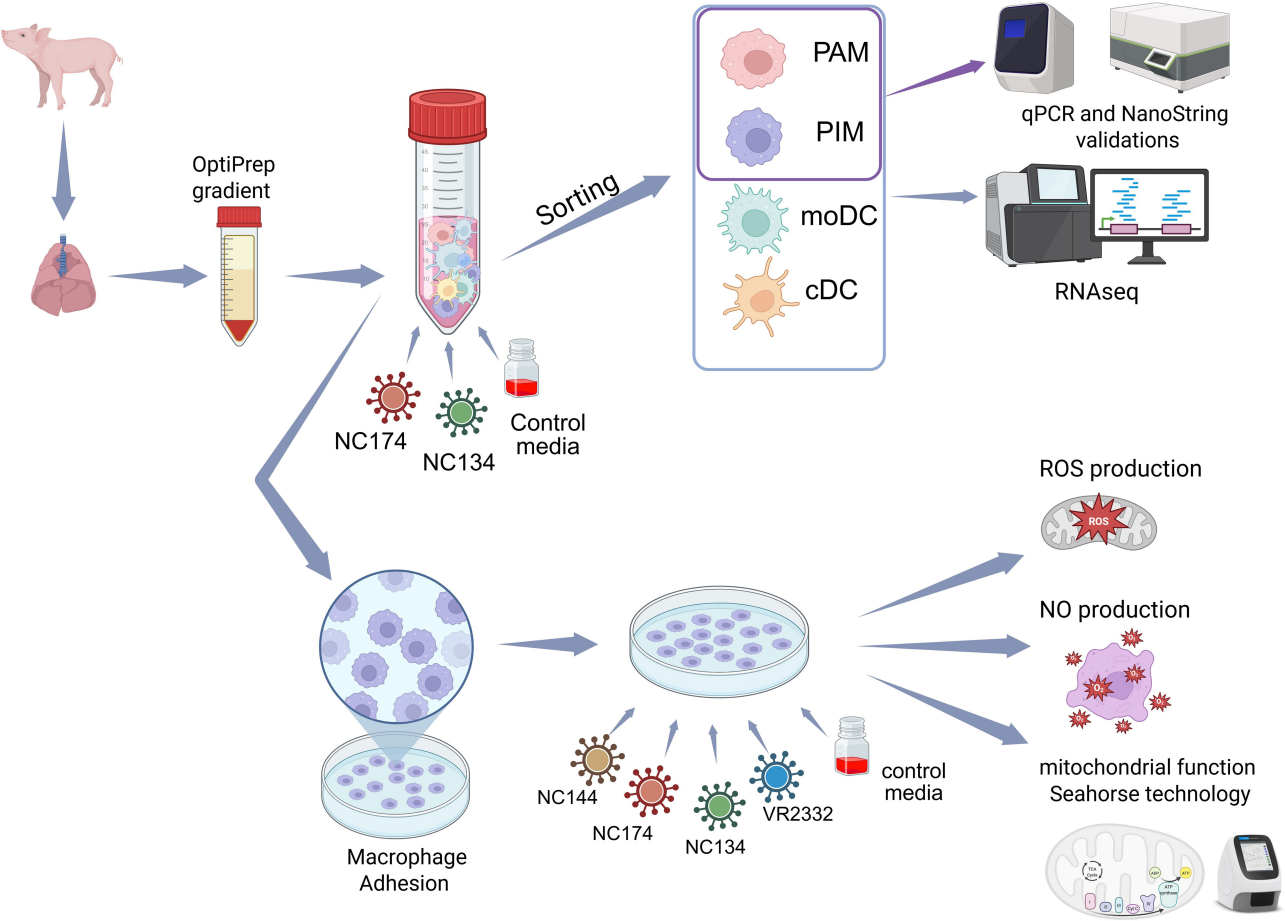
Experimental layout. Lungs were collected from influenza- and PRRSV-negative 8–12-week-old pigs. Mononuclear phagocytes (MNPs) from bronchoalveolar lavage (BAL) and parenchyma (PAR) were enriched using OptiPrep gradient. MNPs were infected with North Carolina (NC) PRRSV-2 strains NC134 and NC174 (MOI = 1) and control media for 12 h. Infected and control porcine alveolar macrophages (PAMs), pulmonary intravascular macrophages (PIMs), monocyte-derived dendritic cells (moDCs), and classical DCs (cDCs) were sorted, and RNA-seq was performed on each cell subset. Additional PAM and PIM sorted cells were used for qPCR and NanoString validations. For other assays, enriched MNPs were plated to allow macrophage adhesion for 2 h. PAMs and PIMs were subsequently infected with different NC PRRSV-2 strains (NC134, NC174, and NC144) or VR2332 prototype strain; uninfected cells were used as negative control. Infected macrophages were used in different assays: mitochondrial function assay (Seahorse Agilent technology) and ROS and NO production assays. Created in BioRender. Crisci, E (2025). https://BioRender.com/65x4hji. PRRSV, porcine reproductive and respiratory syndrome virus; MOI, multiplicity of infection; ROS, reactive oxygen species; NO, nitric oxide.

For functional analyses, PIMs were isolated from enriched PAR MNPs through attachment as previously described (14, 43). Briefly, enriched MNPs were seeded in a culture plate and incubated for 2 h at 37 °C in 5% CO_2_ in complete RPMI. After incubation, the supernatant containing the non-adhered MNPs was removed, leaving PIMs attached to the plate (**Figure 1**).

For the general layout of the performed experiments, PAMs and PIMs were isolated and infected at a multiplicity of infection (MOI) of 1–3 at 37 °C in 5% CO_2_ using different time points depending on each assay.

### 2.2 Viruses

For this study, three North Carolina PRRSV-2 field isolates were used: NC-18-9-7 (GenBank accession ID PP171544, referred to as NC174, lineage 1A. unclassified) (44, 45), NC134 (GenBank accession ID ON844087, L1C.3) (44, 45), and NC20-1 (GenBank accession ID OR805486, referred to as NC144, L1A.17) (46). Additionally, the reference strain VR2332 (ATCC strain BIAH-001, GenBank accession ID U87392.3, L5A.1) was used as the PRRSV-2 prototype strain. Virus stocks were expanded using cultures of PAMs and/or MA-104 cell lines as previously described (44) and stored at −80 °C.

Virus sequences were aligned and compared using the Clustal W algorithm of BioEdit 7.7.1 (https://bioedit.software.informer.com/). Evolutionary divergence and the phylogenetic trees of the nucleotide complete genome and ORF5 were reconstructed with MEGA11 (47) using maximum likelihood with 1,000 bootstrap replicates and the GTR+G nucleotide substitution model (**Table 1**). Moreover, analysis of amino acid sequences on the complete genome and ORF5 was performed using maximum likelihood with 1,000 bootstrap replicates and the JJT+G model amino acid substitution model. The phylogenetic tree for the whole genome nucleotide sequence is available within **Table 1**.

**Table 1:** Percentage identity of virus nucleotide and amino acid sequences.

Genome nucleotide (% identity)	NC134	NC18-9-7 (NC174)	NC20-1 (NC144)	VR2332
NC134				
NC18-9-7 (NC174)	88.5			
NC20-1 (NC144)	87.7	95.6		
VR2332	79.7	79.5	78.6	
				
Genome amino acid (% identity)	NC134	NC18-9-7 (NC174)	NC20-1 (NC144)	VR2332
NC134				
NC18-9-7 (NC174)	76.1			
NC20-1 (NC144)	74.2	91.9		
VR2332	66.5	64.7	63.4	
				
ORF5 nucleotide (% identity)	NC134	NC18-9-7 (NC174)	NC20-1 (NC144)	VR2332
NC134				
NC18-9-7 (NC174)	86			
NC20-1 (NC144)	84.7	85.7		
VR2332	87.3	94.8	86.4	
				
ORF5 amino acid (% identity)	NC134	NC18-9-7 (NC174)	NC20-1 (NC144)	VR2332
NC134				
NC18-9-7 (NC174)	83.5			
NC20-1 (NC144)	82.5	89		
VR2332	84	91.5	89	
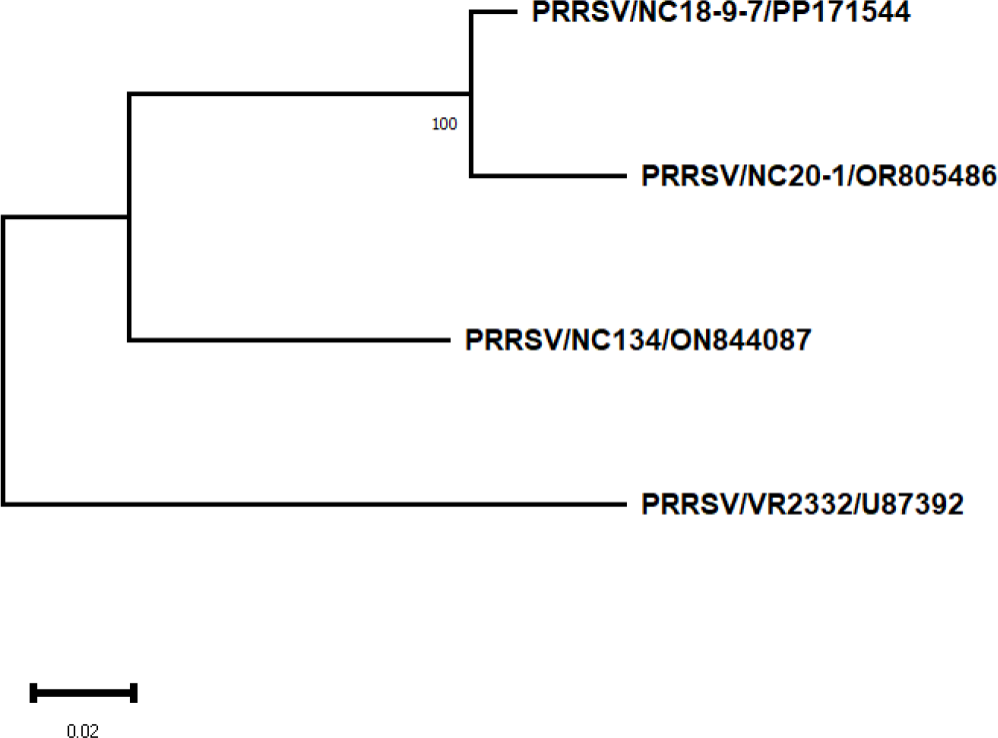				

Phylogenetic tree of whole genome nucleotide sequences.

### 2.3 Sorting

BAL and PAR samples were isolated from three male and three female animals between 8 and 12 weeks of age. For each pig, 30 million enriched MNPs from both BAL and PAR were infected with NC134 and NC174 at MOI = 1 or left uninfected in 50-mL Falcon tubes. Infections were synchronized, and after 12 h, cells were washed and stained with a five-color flow cytometry staining to sort alveolar macrophages (AMs), PIMs, monocyte-derived dendritic cells (moDCs), and classical DCs (cDCs), using a modified sorting strategy from Crisci et al. and Bordet et al. (12, 40).

Briefly, cells were stained in a blocking solution composed of PBS/2 mM EDTA supplemented with 5% FBS. Antibodies (**Table 2**) were added to the blocking solution for 30 minutes on ice and then washed in the blocking solution. Primary antibodies (mouse anti-MHC/SLA-II MSA3, chicken anti-CadM1, and mouse anti-CD172a) and then the secondary isotype and species-specific fluorochrome-coupled antibodies (anti-mouse IgG2a-PE-Cy7, anti-chicken IgG-AF647, and anti-mouse IgG2b-FITC) were used. Then, cells were incubated with the primary antibody anti-CD163-PE, followed by 4′,6-diamidino-2-phenylindole (DAPI) staining to exclude dead cells. After staining, the cells were filtered through a 0.45-µm filter and sorted. Compensation was set according to single-color staining and Fluorescence Minus One (FMO) controls. Sorting efficiency was checked with beads and was at 98%. A Beckman Coulter MoFlo XDP sorter was used to isolate specific cell subpopulations. The FlowJo software (version 10.1.0, Tree Star, Ashland, OR, USA) was used to analyze subpopulation prevalence.

**Table 2:** Antibodies.

Antibody type	Antigen	Clone	Supplier	Catalog #	Isotype	Fluorochrome	Dilution
Primary	MHC II	MSA3	Monoclonal Antibody Center, Washington State University	PG2006	Mouse IgG2a		1/75
Primary	CD172a	74-22-15A	Monoclonal Antibody Center, Washington State University	PG2031	Mouse IgG2b		1/150
Primary	Anti-SynCAM (TSLC1/CADM1)	30	MBL International	CM004-3	Chicken IgY		1/150
Primary	CD163	2A 10/11	Invitrogen	MA5-16476	Mouse IgG1	PE	1/30
Secondary	Rat Anti-Mouse IgG2a	m2a-15F8	Invitrogen	25-4210-82		PE-Cyanine7	1/100
Secondary	Rat Anti-Mouse IgG2b	m2b-25G4	Invitrogen	11-4220-82		FITC	1/100
Secondary	Goat Anti-Chicken IgG (H+L) (polyclonal)	Polyclonal	Invitrogen	A21449		Alexa Fluor 647	1/100
DAPI			Invitrogen	D1306			1/1,000

PAMs were sorted from BAL samples, and PIMs, cDCs, and moDCs were sorted from PAR samples for each condition. We focused the sorting on PAM and PIM subpopulations (sorting strategy for macrophages in **Supplementary Figure 1**), and the other MNPs were used to confirm the identities of the cell subsets using a cross-species transcriptional signature enrichment analysis. After sorting, approximately 300,000 PAMs, 100,000 PIMs, 64,000–100,000 moDCs, and 63,000–100,000 cDCs were collected for each condition.

### 2.4 RNA sequencing

Total RNA was obtained from approximately 64,000 to 100,000 sorted MNPs of four pigs (two females and two males) using the PicoPure RNA Isolation Kit (Thermo Fisher, KIT0204) following the manufacturer’s instructions.

RNA quantity was determined using the NanoDrop 1000 (Thermo Fisher) and an Agilent Bioanalyzer (Agilent, Santa Clara, CA, USA), with RNA Integrity Number (RIN) values ranging from 7.1 to 9.6 and concentrations ranging from 0.11 to 21.61 ng/μL. RNA-seq libraries were generated from 1 ng of total RNA per sample using a NEBNext Single Cell/Low Input RNA Library Prep Kit for Illumina (New England Biolabs, Ipswich, MA, USA) and NEBNext Multiplex Oligos for Illumina (New England Biolabs, Ipswich, MA, USA) following the manufacturer’s instructions. Amplified cDNA quality and quantity were assessed using the Agilent Technologies 2200 TapeStation with the High Sensitivity D5000 tape. For library preparation, 2 ng of amplified cDNA per sample was used. Library quality was assessed using the Agilent Technologies 2200 TapeStation with the D1000 tape. Libraries were quantified by RT-qPCR using the NGS Library Quantification kit (Takara Bio, San Jose, CA, USA) following the manufacturer’s instructions. A total of 47 cDNA libraries were diluted to 10 nM, pooled, and submitted to Novogene for 150-bp paired-end sequencing on a NovaSeq 6000 S4 lane (Illumina).

### 2.5 RNA-seq data analysis

Sample raw reads were mapped to an indexed swine reference genome (Ensembl Sscrofa11.1), and transcript abundances were quantified using Kallisto (v0.46.2). The quality of the reads and the results of Kallisto mapping were assessed using fastqc and multiqc. Data pre-processing and analysis were performed using R (v4.4.2) for all samples together and also separately for PAM and PIM samples. Gene annotation was retrieved using the BiomaRt package (2.62.1 with Ensembl genes v110). After trimmed mean of M values (TMM) normalization, hierarchical clustering as well as principal component analyses revealed a dominant batch effect linked to animals or harvesting days. This batch effect was then corrected using the ComBat-seq package applied to raw transcript counts (48). Differential gene expression analysis was performed using the edgeR package.

High-throughput Gene Set Enrichment Analysis (GSEA) was applied using BubbleMap, a module of the BubbleGUM software (49), with transcriptional signatures of murine MNP cells [Methods, Results, and Supplementary Material from (12)] to validate the sorting strategy. BubbleMap was also applied with the Hallmark gene set from MSigDB and with the genes found significantly up- or downregulated in PIMs or PAMs upon viral infection in order to gain insights into the transcriptional changes undergone by the cells.

Functional annotation enrichment analysis was performed on the differentially expressed genes (DEGs) using the enrichR package, querying different databases, including Gene Ontology (biological processes) and MSigDB (Hallmark) (50).

### 2.6 NanoString

NanoString experiments were performed on additional samples of PAM and PIM sorted cells (as described in Section 2.3) infected at MOI = 1 for 12 h. For the analysis, a custom CodeSet of 46 genes was used (**Supplementary Table 1**). The NanoString panel was designed to incorporate a broad spectrum of immune genes relevant to inflammatory and antiviral responses, oxidative stress, myeloid cell activation, apoptosis, receptor signaling, and the complement system.

Total RNA was extracted using the RNeasy kit (Qiagen, Germantown, Maryland, USA) following the manufacturer’s instructions, and purity and concentration were determined using NanoDrop 1000 (Thermo Fisher). Gene expression was measured using the NanoString nCounter^®^ analysis system. Briefly, RNA was hybridized with biotin-labeled capture probes and fluorescently labeled reporter probes for 18 h at 65 °C following the manufacturer’s instructions (NanoString Inc., Seattle, WA, USA; MAN-C0035-07). Following hybridization, samples were transferred into a NanoString cartridge and loaded onto the nCounter Prep Station instrument set on high sensitivity. Then, the cartridge was transferred into the nCounter Digital Analyzer, where excess capture and reporter probes were removed, and hybridized RNA was immobilized for imaging. The cartridge was then scanned using the nCounter Digital Analyzer (NanoString Technologies, Seattle, WA, USA) at 555 field of view resolution. Following image acquisition, RNA counts were analyzed and exported using the nSolver analysis software (nSolver 4.0 Analysis Software, Inc., Seattle, WA, USA). Samples that failed Quality Control (QC) were excluded from downstream analysis. Background levels were calculated as geometric means of negative controls ± 2* standard deviation (SD), and expression data below the background were considered negative. Gene expression counts were normalized to housekeeping genes.

### 2.7 RT-qPCR

Additional PAM and PIM sorted cells and cDNA remaining from library preparation were used for RT-qPCR. Samples were lysed, and RNA was extracted using the PicoPure RNA Isolation Kit (Thermo Fisher) following the manufacturer’s instructions. cDNA synthesis was performed using the iScript™ cDNA Synthesis Kit (Bio-Rad, Hercules, CA, USA), or cDNA from library preparation was used (Section 2.4). The primers used for the detection of the genomic copies of PRRSV-2 strains were nsp9 F (5′-CCTGCAATTGTCCGCTGGTTTG-3′) and nsp9 R (5′-GACGACAGGCCACCTCTCTTAG-3′), previously described by Spear and Faaberg (51). Additional primers were IL1B F (5′-TGCCAACGTGCAGTCTATGG-3′) and R (5′-TGGGCCAGCCAGCACTAG-3′) (52), IFNB F (5′-TGTGGAACTTGATGGGCAGA-3′) and R (5′-GAATGGTCATGTCTCCCCTGG-3′), and RPS24 F (5′-AAGGAACGCAAGAACAGAATGAA-3′) and R (5′-TTTGCCAGCACCAACGTTG-3′) as a housekeeping gene (40). All primers used in this study were purchased from Integrated DNA Technologies, Research Triangle Park, North Carolina, USA. To reduce the variation between samples in the RT-qPCR raw data, the Ct values of the housekeeping gene were used to perform a ΔCt normalization. Subsequent normalization to the mean of the mock values was performed for the expression of IL1B and IFNB.

### 2.8 Mitochondrial function

Mitochondrial function of lung macrophages was measured using the Seahorse Cell Mito Stress assay (Agilent, Santa Clara, CA, USA). First, PAMs and PIMs were isolated as previously described in Section 2.1, seeded in a culture cartridge at a density of 150,000 cells per well, and infected with PRRSV-2 at MOI = 1 overnight (12–16 h). Cells left untreated were used as control cells. On the day of the assay, Seahorse media, detection cartridge, and the three drugs provided in the kit [oligomycin, carbonyl cyanide-4-(trifluoromethoxy)phenylhydrazone (FCCP), and rotenone/antimycin A (AA)] were prepared following the manufacturer’s instructions. The final drug concentration in the well was 1.5 µM for oligomycin, 2 µM for FCCP, and 0.5 µM for rotenone/AA. Measurements were performed using the Seahorse XFp analyzer (Agilent, Santa Clara, CA, USA) available at the NCSU College of Veterinary Medicine core facility. Basal respiration, maximal respiration, spare respiratory capacity, non-mitochondrial oxygen consumption, ATP-linked respiration, and coupling efficiency % were evaluated using the Wave software (Agilent). Cell respiratory control ratio (RCR) and the ratio of ATP-linked respiration to maximal respiration were calculated as described in Divakaruni et al. (53).

### 2.9 ROS and NO production

To measure ROS production, PAMs and PIMs were seeded in black-wall 96-well plates (Greiner Bio-One, Monroe, North Carolina, USA) at a density of 150,000 cells per well, infected with PRRSV at MOI = 1–3, or left untreated (mock). Dihydrorhodamine 123 (excitation, 485/20; emission, 528/20) was added into the wells at a concentration of 10 µM following the manufacturer’s instructions (Chemodex, St. Gallen, Switzerland). Plates were incubated at 37 °C in a 5% CO_2_ incubator protected from light, and fluorescence was measured using the BioTek Synergy 2 plate reader at different time points (12, 24, 48, and 72 h). Results are shown as relative fluorescence units (RFU).

The measurement of nitrite concentration as an index of nitric oxide (NO) production was analyzed using the Griess reaction system following the manufacturer’s instructions (Promega, Madison, WI, USA). Briefly, 50 µL of supernatant from the samples was used in parallel with a standard curve. The content was treated with sulfanilamide (SA) first for 10 minutes and then with *N*-naphthyl-ethylenediamine (NED) for an additional 10 minutes to form a stable azo compound. Absorbance was measured at 540 nm and was proportionally correlated to the standard, ranging from 0 to 100 µM.

### 2.10 IFN-α ELISA

Supernatants from BAL MNPs and PAR MNPs infected and control cultures used for the sorting were examined for IFN-α using capture ELISA at 12 h post-infection. Anti-IFN-α monoclonal antibodies (K9 and K17) and an IFN-α recombinant protein (PBL Biomedical Laboratories, Piscataway, NJ, USA) were used in ELISA as previously described (54). Data are shown in IU/mL.

## 3 Results

A general illustration of the experimental layout can be found in **Figure 1**. Briefly, macrophages were sorted or obtained and isolated as described in the Methods section and infected with PRRSV-2 at different MOIs and time points or left untreated. Then, cells were used in four main assays: gene expression analyses (RNA-seq, NanoString, and RT-qPCR), evaluation of mitochondrial function using Seahorse technology, and assays for ROS and NO production.

### 3.1 Sorting of lung MNP cells

To confirm the identities of the cell subsets sorted using our strategy, we performed a cross-species transcriptional signature enrichment analysis using high-throughput GSEA with the BubbleGUM software, and we projected the results as a BubbleMap. We used the signatures of murine MNP subsets as gene sets, and we compared them with the swine samples from the mock condition as previously performed (Methods, Results, and Supplementary Material sections from Crisci et al. (12)). The murine gene sets included splenic and/or lung cDC1, cDC2, DCs vs. macrophages, lung macrophages, and monocyte-derived macrophage signatures.

The cross-species transcriptional analysis confirmed that genes highly expressed in murine DCs, as well as the splenic murine cDC2 signatures and splenic and lung cDC1 signatures—to a lesser extent—were enriched in swine lung cDCs when compared to all other cell subsets (**Supplementary Figure S2A**, green square). As expected, these cells expressed higher levels of cDC markers such as FLT3, BCL11A, MSI2, CADM1, and BATF3 (**Supplementary Figure S2B**). Conversely, the genes more highly expressed in murine macrophages as compared to murine DCs were enriched in swine PAMs, PIMs, and MoDCs when compared to swine cDCs (**Supplementary Figure S2A**, purple square). The murine lung macrophage signature was also found enriched in swine PAMs and PIMs when compared to swine cDCs and moDCs and was found enriched in PAMs when compared to PIMs (**Supplementary Figure S2A**, orange and turquoise squares). Consistent with this BubbleMap result, sorted PAMs and PIMs expressed a high level of macrophage genes such as CEBPB, CD163, MERTK, FCGR1A, and MRC1 (**Supplementary Figure S2B**). The murine lung monocyte-derived macrophage signature was found enriched in swine sorted moDCs when compared to all three other cell subsets, although not at a significant level (**Supplementary Figure S2A**, brown square), thus confirming the monocyte origin of these cells. Sorted moDCs also expressed a higher level of known moDC markers, such as CSF1R and IL1R2 (**Supplementary Figure S2B**), hence supporting their identity.

Overall, these results validate the identity of our uninfected sorted cell populations, especially the lung macrophage identity of the PAM and PIM subsets.

To gain more insights into the similarities vs. differences between MNP subpopulations, including the relative impacts of cell types and infection status, we performed dimensionality reduction using principal component analysis (PCA) of the porcine lung cell transcriptional profiles with or without *in vitro* stimulation with the two viral strains, NC134 and NC174. The approach revealed a clear segregation on PC1 between lung tissue macrophages and cDCs, as previously described in Crisci et al. (12) (**Supplementary Figure S2C**). This analysis confirmed that moDCs segregate with lung macrophages and away from cDCs on PC1 (31.3% of total variance), confirming their closer proximity to lung macrophages and their relative difference with cDCs as seen in Crisci et al. (12). PC2 (13% of total variance) separated the lung macrophages on one side and moDCs on the other, with PIMs being located between PAMs and moDCs (**Supplementary Figure S2C**). Interestingly, PC3 and PC4 (contributing only to 6.6% and 4% of total variance, respectively) were not associated with the response to viruses (**Supplementary Figure S2D**), hence showing that the impact of the infection status was overweighed by the cell types, thus indicating that it is necessary to analyze each cell type independently to gain insights into the response to viruses.

### 3.2 Transcriptomic signatures of PAMs and PIMs upon PRRSV-2 infection with NC174 and NC134

**Figure 2:**
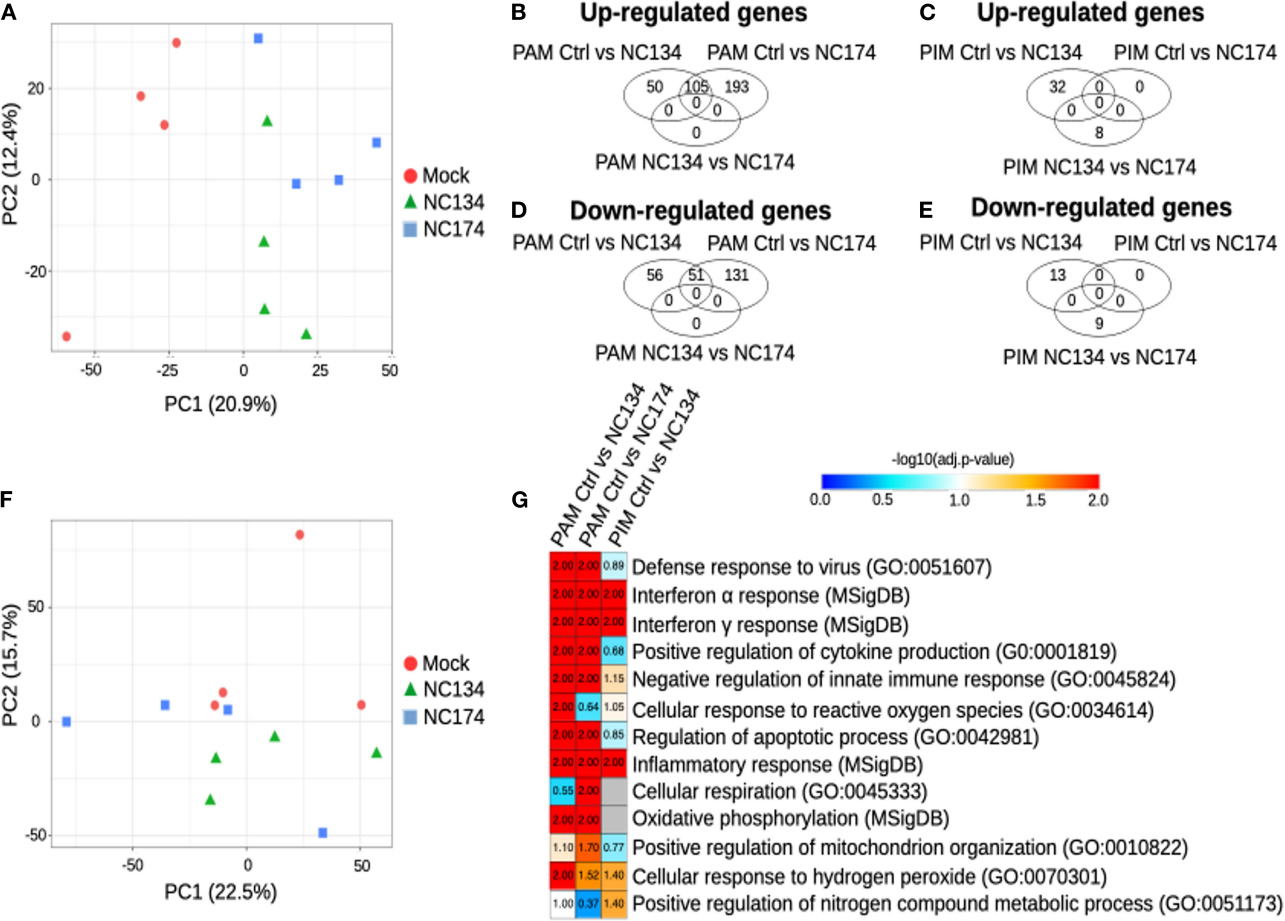
Cell type-specific responses of PAMs and PIMs to PRRSV-2 infection. RNA-seq analysis. **(A)** Principal component analysis of the PAM samples, PC1 vs. PC2. **(B)** Venn diagram showing the genes found upregulated in PAMs, with FDR < 0.05. **(C)** Venn diagram showing the genes found upregulated in PIMs, with FDR < 0.05. **(D)** Venn diagram showing the genes found downregulated in PAMs, with FDR < 0.05. **(E)** Venn diagram showing the genes found downregulated in PIMs, with FDR < 0.05. **(F)** Principal component analysis of the PIM samples, PC1 vs. PC2. **(G)** Functional annotation enrichment analysis. Heatmap showing selected functions found enriched in the MNPs infected with NC134 or NC174 as compared to control (Mock). Data represent four biological replicates for all the conditions. PAMs, porcine alveolar macrophages; PIMs, pulmonary intravascular macrophages; PRRSV, porcine reproductive and respiratory syndrome virus; NC, North Carolina; MNPs, mononuclear phagocytes.

Using the RNA-seq output (Sections 2.4 and 2.5), we focused on the lung macrophages with the aim of characterizing their cell-specific responses to PRRSV-2 exposure and comparing the transcriptional reprogramming linked to the NC174 versus the NC134 strain. We analyzed the PIM and PAM datasets separately and compared their responses to each strain. Dimensionality reduction using PCA revealed a stronger impact of PRRSV-2 infection on the reprogramming of PAMs as compared to PIMs. Infected PAMs segregated away from mock PAMs along PC1, whereas such separation is barely observed between mock and infected PIMs (**Figures 2A, F**). This stronger impact on PAMs rather than PIMs is confirmed by a higher number of statistically significant DEGs in infected PAMs (262 DEGs upon NC134 infection, 480 DEGs upon NC174 infection, and 156 in common; **Figures 2B, D**, **Supplementary Table 2**) as compared to infected PIMs (45 DEGs upon NC134 infection and 0 DEGs upon NC174 infection; **Figures 2C, E**, **Supplementary Table 2**). However, we did not observe any clear difference between NC134 and NC174 infections in PAMs or PIMs (0 DEGs in PAMs and 17 DEGs in PIMs; **Figures 2B–E**).

Next, we performed functional annotation enrichment analyses on the DEGs to obtain insights into the pathways altered in PAMs or PIMs upon PRRSV-2 infection. In PAMs, upon infections of either NC134 and NC174, pathways found significantly enriched included the following: defense response to viruses (False Discovery Rate (FDR) < 0.01), responses to Type I (FDR < 0.01) and Type II IFN (FDR < 0.01), positive regulation of cytokine production (FDR < 0.01), negative regulation of innate immune responses (FDR < 0.01), regulation of apoptotic process (FDR < 0.01), inflammatory responses (FDR < 0.01), oxidative phosphorylation (FDR < 0.01), and cellular response to hydrogen peroxide (FDR < 0.05 and FDR = 0.03, respectively) (**Figure 2G**, **Supplementary Table 3**). Cellular response to ROS was found significant only upon NC134 infection (FDR = 0.01), while cellular respiration and positive regulation of mitochondrion organization were found significant only upon NC174 infection (FDR < 0.05 and FDR = 0.02, respectively).

DEGs found in PIMs upon PRRSV-2 infection were enriched for pathways in common with some found in PAMs, including interferon alpha and gamma response (FDR = 0.01), cellular response to hydrogen peroxide (FDR = 0.04), and inflammatory response (FDR < 0.01), but also for positive regulation of nitrogen compound metabolic process (FDR = 0.04) (**Figure 2G**, **Supplementary Table 3**). More generally, it appears that the global transcriptional reprogramming undergone by PIMs in response to PRRSV-2 infection is more subtle than that undergone by PAMs in terms of both DEG numbers and altered pathways.

**Figure 3:**
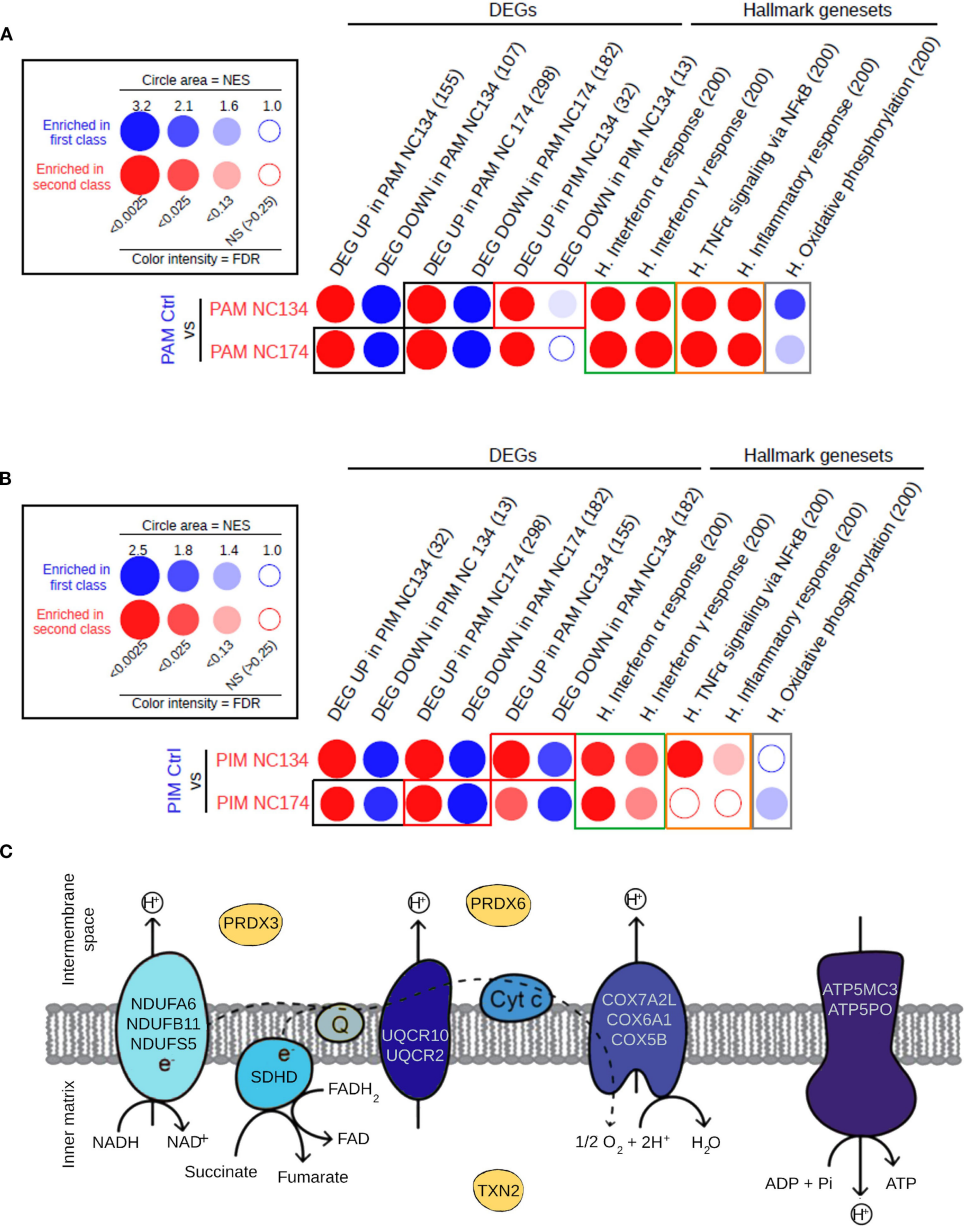
Similarity in the responses of PIMs and PAMs to PRRSV-2 infection. **(A)** Analysis of the pathways and gene sets of interest enriched in PAMs upon NC134 and NC174 infections by high-throughput GSEA using BubbleGUM. **(B)** Analysis of the pathways and gene sets of interest enriched in PIMs upon NC134 and NC174 infections using BubbleGUM. The Hallmark gene sets from MSigDB (H.), together with lists of DEGs in PAMs or PIMs in response to NC134 or NC174 infection, were used to assess their enrichments in the comparisons between macrophage subsets in control (Mock) vs. NC134 or NC174 conditions using the BubbleMap module of BubbleGUM. Gene numbers per gene set are written in parentheses. **(C)** Genes located in the mitochondrial membrane that are enriched in the mitochondrial dysfunction pathway or in the downregulation of the oxidative phosphorylation in NC174-infected PAMs. Data represent four biological replicates for all the conditions. PAMs, porcine alveolar macrophages; PIMs, pulmonary intravascular macrophages; PRRSV, porcine reproductive and respiratory syndrome virus; GSEA, Gene Set Enrichment Analysis; DEGs, differentially expressed genes.

In order to complete this analysis, which was limited by the low number of DEGs in PIMs upon NC134 and NC174 infections (45 and 0, respectively), we applied another GSEA-based analysis using BubbleGUM, which included all the genes, not only the DEGs, and detected the statistically relevant enrichment of the gene sets of interest. The similarity of responses in both PIMs and PAMs was confirmed using BubbleMap. Pathways including IFNA and IFNG responses were shown to be significantly upregulated in both PAMs and PIMs upon NC134 or NC174 infection (**Figures 3A, B**, green squares). TNFA signaling via NF-κB and inflammatory response were also found upregulated in both PAMs and PIMs upon NC134 or NC174 infection, although not reaching our statistical cut-off for PIMs upon NC174 infection (**Figures 3A, B**, orange squares). Conversely, oxidative phosphorylation was found to be downregulated upon PRRSV-2 infection in both PAMs and PIMs, although not significant in PIMs upon NC134 infection (**Figures 3A, B**, gray squares). These results, based on the analysis of all the genes, confirm what was already observed on the DEGs in PAMs and show that PIMs, in response to PRRSV-2 infection, tend to regulate the same pathways as PAMs, but to a lesser extent. To further compare the responses between PIMs and PAMs upon NC134 or NC174 infection, we used as gene sets the DEGs found for each lung macrophage upon each strain infection. The DEGs found both up- and downregulated in PAMs upon NC134 infection were also found significantly up- and downregulated, respectively, in PAMs infected with NC174, and vice versa (**Figure 3A**, black squares). The DEGs found up- and downregulated in PIMs upon NC134 infection were also found significantly up- and downregulated, respectively, in PIMs infected with NC174 (**Figure 3B**, black squares). DEGs found up- and downregulated in NC134 PIMs were also found significantly up- and downregulated, respectively, in NC134 PAMs (**Figure 3A**, red square), and DEGs found up- and downregulated in PAMs upon NC134 or NC174 infection were also found significantly up- and downregulated, respectively, in PIMs infected with NC134 or NC174, respectively (**Figure 3B**, red squares).

These results confirm the similar reprogramming for both NC134 and NC174 infections, in either PAMs or PIMs, thus suggesting a similar virulence of the two viral strains.

When the specific downregulated DEGs related to mitochondrial function and oxidative phosphorylation were further analyzed in PAMs infected with NC174, we observed that the majority were linked to the different complexes of the mitochondrial electron transport chain, except for the three genes: PRDX3 and PRDX6 enzymes involved in controlling cellular redox state and oxidative stress (55, 56), and TXN2, expressed in the inner mitochondrial matrix, which also encodes a protein involved in controlling redox and protecting against oxidative stress (57) (**Figure 3C**).

These findings highlight a multifaceted disruption of mitochondrial homeostasis in PAMs upon PRRSV-2 infection, impacting both energy production and oxidative stress management.

### 3.3 NanoString, RT-qPCR, and IFN-α production validations for lung macrophages

NanoString and RT-qPCR were used for further validation of PAM and PIM transcriptional changes upon PRRSV-2 infection observed by RNA-seq (**Supplementary Figures 3A, B**). There was a positive correlation between RT-qPCR, NanoString, and RNA-seq: Spearman’s correlation coefficient of ρ = 0.9904 in PAMs, ρ = 0.9858 in PIMs between NanoString and RT-qPCR, ρ = 0.8544 in PAM mock, ρ = 0.882 in PAM NC134, and ρ = 0.814 in PAM NC174 between NanoString and RNA-seq data.

Additionally, RT-qPCR was performed on the additional duplicate PAM and PIM samples or on samples used for RNA-seq and NanoString to detect the levels of RNA virus present in sorted cells. As expected, sorted PRRSV-2-infected cells showed PRRSV NSP9 expression, and the level of infection was similar between NC134 and NC174 in PAMs and PIMs (**Supplementary Figure 3B**). This was also consistent with their similar growth kinetics observed *in vitro* at 24 and 48 h post-infection (**Supplementary Figure 3C**).

The expression of IFNB and IL1B was significantly higher in infected cells compared with mock, but no significant differences were found between NC134 and NC174 strains in PAMs and PIMs (**Supplementary Figure 3D**).

When IFN-α protein production was evaluated using ELISA, infected BAL MNPs showed significantly higher levels compared to control cells, with no difference between NC134 and NC174. However, PAR MNP infected cells did not show any significant difference in IFN-α production compared to control cells (**Supplementary Figure 3E**).

### 3.4 PRRSV-2 infection impairs the mitochondrial fitness of lung macrophages

Based on the bioinformatics analyses highlighting mitochondrial dysfunction associated with PAM and PIM infection, we examined those organelles through functional analyses, testing the mitochondrial fitness of the cells.

The Seahorse Cell Mito Stress assay measures mitochondrial function via oxygen consumption rate (OCR) of live cells in real time, providing information about basal and maximal respiration, ATP-linked respiration, and spare respiratory capacity. Spare respiratory capacity indicates the capability of the cell to respond to an energetic demand as well as how close the cell is to respire to its theoretical maximum. Additional metabolic analyses that are independent of the cell number were performed: the coupling efficiency %, which can estimate the fraction of basal respiration used to drive ATP synthesis; the cell respiratory control ratio; and the ratio of ATP-linked respiration and maximal respiration, which can estimate how oxidative phosphorylation is making ATP relative to its maximum capacity (53).

**Figure 4:**
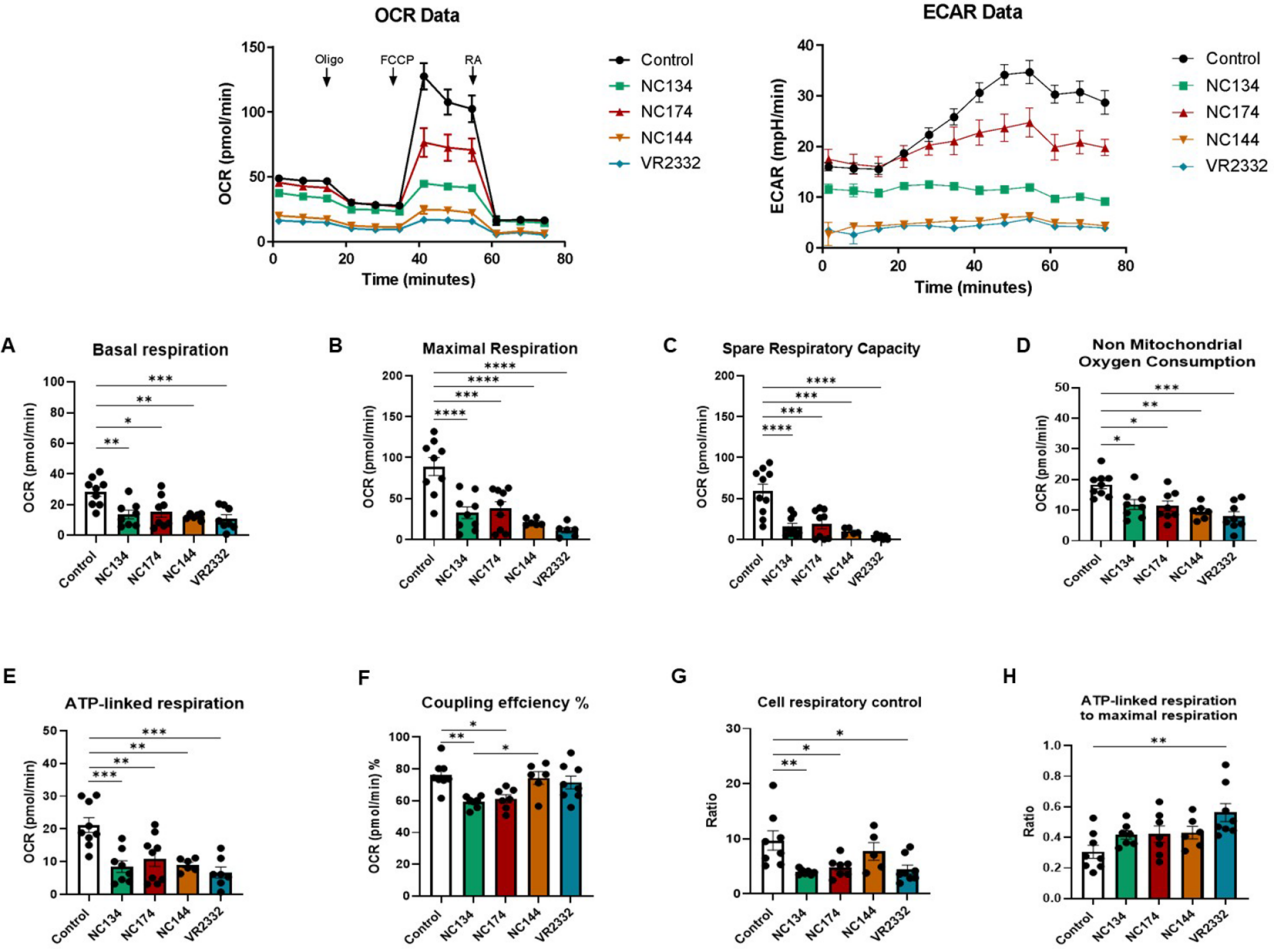
Mitochondrial dysfunction in PRRSV-2-infected porcine alveolar macrophages (PAMs). PAMs were infected for 12–16 h with different PRRSV-2 strains and washed, and mitochondrial dysfunction was measured using the Cell Mito Stress Test kit (Agilent, 103010-100). Top figures correspond to representative figures of the key components of mitochondrial respiration in the Seahorse Cell Mito Stress assay, including oxygen consumption rate (OCR) (Left) profiles and extracellular acidification rate (ECAR) (Right) in infected and control PAMs. Oligomycin (Oligo) reduces the oxygen consumption ratio (OCR) through inhibition of ATP synthase, a critical facilitator of oxidative phosphorylation of ADP into ATP. Carbonyl cyanide-4-(trifluoromethoxy)phenylhydrazone (FCCP) potently collapses the proton gradient at the inner mitochondrial membrane, simulating a maximal energy demand in the cells. Rotenone and antimycin A are complex I and complex III inhibitors, respectively, of the electron transport chain (ETC). The profile of mitochondrial respiration in the test includes basal respiration **(A)**, maximal respiratory capacity **(B)**, spare capacity **(C)**, non-mitochondrial oxygen consumption **(D)**, ATP-linked respiration **(E)**, and coupling efficiency **(F)**. **(G)** Cell respiratory control. **(H)** ATP-linked respiration to the maximal respiration as described in Divakaruni et al. (53). N = 7–9. Data were analyzed in Wave software (Agilent) and visualized using GraphPad Prism v10. Statistics: ordinary one-way ANOVA. *p < 0.05, **p < 0.01, ***p < 0.001, and ****p < 0.0001. PRRSV, porcine reproductive and respiratory syndrome virus.

Seahorse analyses showed different profiles in both cell types. In the case of PAMs (**Figure 4**), all the PRRSV-2 strains induced a decrease in basal respiration and maximal respiration compared to mock (p < 0.05) (**Figures 4A, B**). All strains reduced the spare respiratory capacity, meaning the capability of the cell to respond to an energetic demand and indicating cell fitness and flexibility (**Figure 4C**). Additionally, all strains influenced non-mitochondrial oxygen consumption and ATP-linked respiration (**Figures 4D, E**). In the case of coupling efficiency and cell respiratory control, NC134 and NC174 showed a significant decrease compared to the other strains (**Figures 4F, G**). VR2332 showed a significant decrease in cell respiratory control compared to uninfected cells and was the only strain with a significant increase in the ratio of ATP-linked respiration to maximal respiration compared to control cells (**Figures 4G, H**).

**Figure 5:**
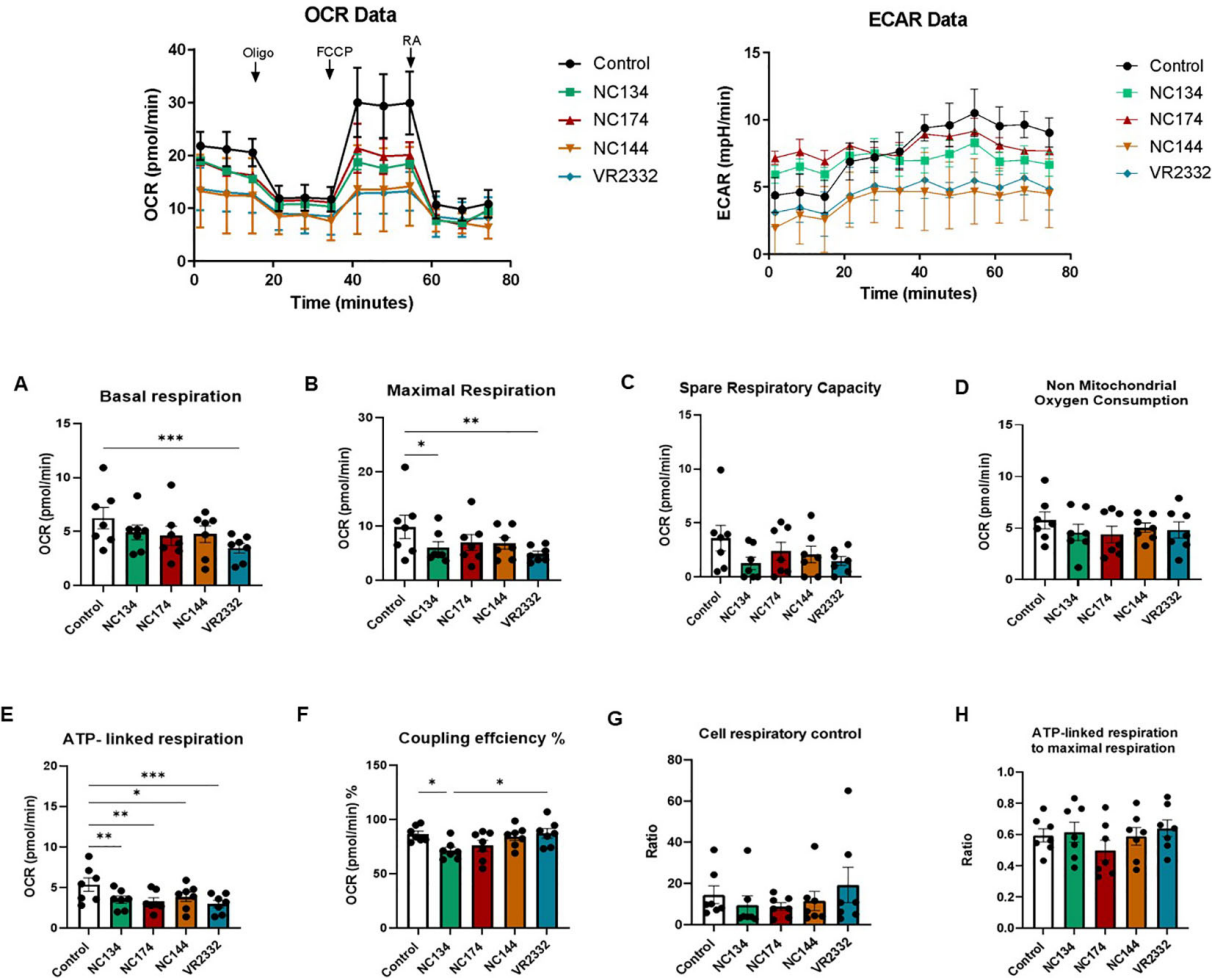
Mitochondrial dysfunction in PRRSV-2-infected pulmonary intravascular macrophages (PIMs). After enriched parenchyma cell adhesion for 2 h and removal of non-adherent cells, PIMs were infected for 12–16 h with different PRRSV-2 strains and washed, and mitochondrial dysfunction was measured using the Cell Mito Stress Test kit (Agilent, 103010-100). Top figures correspond to representative figures of the key components of mitochondrial respiration in the Seahorse Cell Mito Stress assay, including oxygen consumption rate (OCR) (Left) profiles and extracellular acidification rate (ECAR) (Right) in infected and control PAMs. Oligomycin (Oligo) reduces the oxygen consumption ratio (OCR) through inhibition of ATP synthase, a critical facilitator of oxidative phosphorylation of ADP into ATP. Carbonyl cyanide-4-(trifluoromethoxy)phenylhydrazone (FCCP) potently collapses the proton gradient at the inner mitochondrial membrane, simulating a maximal energy demand in the cells. Rotenone and antimycin A are complex I and complex III inhibitors, respectively, of the electron transport chain (ETC). The profile of mitochondrial respiration in the test includes basal respiration **(A)**, maximal respiratory capacity **(B)**, spare capacity **(C)**, non-mitochondrial oxygen consumption **(D)**, ATP-linked respiration **(E)**, and coupling efficiency **(F)**. **(G)** Cell respiratory control. **(H)** ATP-linked respiration to the maximal respiration as described in Divakaruni et al. (53). N = 7. Data were analyzed in Wave software (Agilent) and visualized using GraphPad Prism v10. Statistics: ordinary one-way ANOVA. *p < 0.05, **p < 0.01, and ***p < 0.001. PRRSV, porcine reproductive and respiratory syndrome virus.

In the case of PIMs, all PRRSV-2 strains significantly impaired ATP-linked respiration, but only a few of them were capable of modulating other metabolic parameters: NC134 and VR2332 showed the lowest maximal respiration levels, with NC134 additionally affecting the coupling efficiency % compared to control cells. Moreover, VR2332 significantly impaired basal respiration compared with uninfected cells (**Figure 5**).

Seahorse functional assays showed the same patterns when experiments were performed using frozen or fresh cells (**Figures 4**, **5**, **Supplementary Figure 4**).

### 3.5 PRRSV-2 infection did not stimulate ROS and NO production in lung macrophages

**Figure 6:**
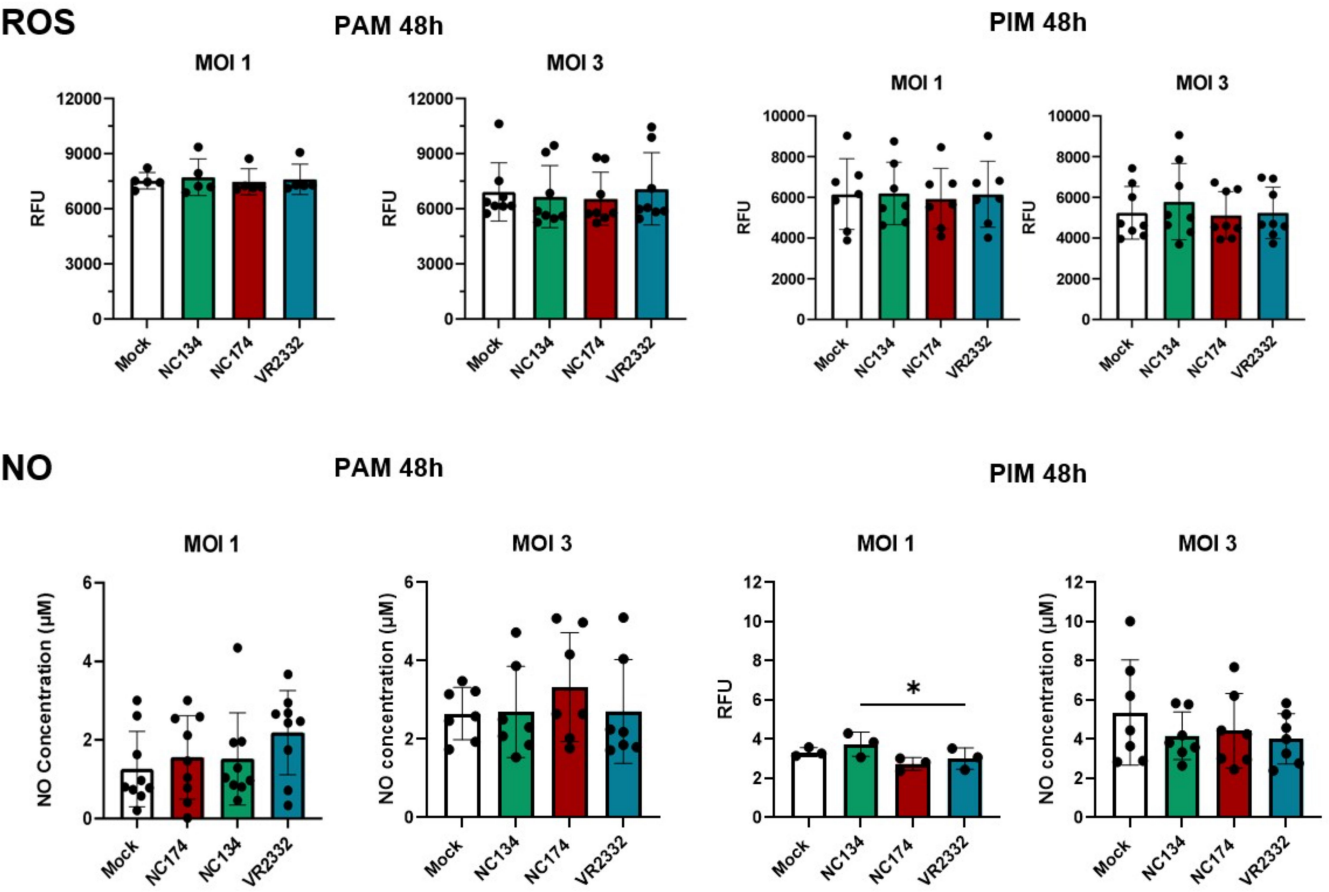
Reactive oxygen species (ROS) and nitric oxide (NO) production in PRRSV-2-infected macrophages. Macrophages were plated and infected with different strains of PRRSV-2. ROS production was measured using DHR-123 from 0 to 72 h. Figures show ROS production at 48 h post-infection with different MOIs (1 and 3). Data are expressed in relative fluorescence units (RFU) measured at 540 nm. N = 5–8. Results were analyzed using one-way ordinary ANOVA with Tukey’s multiple-comparisons test and visualized using GraphPad Prism v10. The measurement of nitrite concentration as an index of nitric oxide (NO) production was analyzed using Griess reaction system following manufacturer’s instructions (Promega). Absorbance was measured at 540 nm and proportionally correlated to the standard, ranging from 0 to 100 µM. N = 3–9. Statistics: ordinary one-way ANOVA. *p < 0.05. PRRSV, porcine reproductive and respiratory syndrome virus; MOIs, multiplicities of infection.

When ROS and NO productions were evaluated at different time points and MOI = 1 and 3, no significant difference was observed between infected macrophages and controls. Both PAMs and PIMs presented the same patterns for ROS and NO at 24 h (data not shown) and 48 h (**Figure 6**) post-infection at MOI = 1 and 3.

## 4 Discussion

Our study provides a comprehensive investigation into the impact of different North Carolina (NC) PRRSV-2 strains (NC134, NC174, and NC144) and the reference strain VR2332 on the metabolic and immune profiles of primary lung macrophages (PAMs and PIMs) during the early stages of infection.

The pathology, infection kinetics, and adaptive immune responses of NC134 and NC174 strains have been previously reported by Kick et al. (44, 45). Both NC134 and NC174 strains were defined as low pathogenic and highly pathogenic, respectively, based on pathogenicity observed during field outbreaks. Nevertheless, there were no significant differences in the clinical signs observed during experimental conditions (e.g., fever, lethargy, and reduced body weight), which could vary with the presence of other pathogens and secondary infections in less controlled field settings (44). In the experimental trial with NC strains, these infections led to a similar viremia timeline and persistence in the lymphoid tissue, with viremia clearance achieved at 49 days post-inoculation (dpi) for NC134 and 35 dpi for NC174 (44). T-cell proliferation was detectable at 14 dpi, peaking at 28 dpi. CD4+ T-helper (Th) cells were the main responders at the first peak. PRRSV-2 NC174 infection also led to a second peak in T-cell proliferation at 56 dpi, which was explained by the high proliferation of TCR-γδ T cells. Those cells dominated the T-cell response in all PRRSV-2 groups at the later time points between 42 and 56 dpi (44). Infection with both NC134 and NC174 strains caused the development of serum neutralizing antibodies within 2 weeks, and NC174 led to a biphasic viremia along with a biphasic curve of IgA production not observed for NC134 (45).

Similar to the experimental trial and the adaptive immunity outcomes, lung macrophage responses evaluated in this study did not present major strain-related differences, especially in the functional assays. Although NC134 and NC174 belong to the same lineage 1, but different sub-lineages [based on ORF5 classification (2, 4, 5)], and their genetic nucleotide homology is below 90%, macrophage transcriptomic signatures were not different, with both strains showing similar patterns of gene modulation compared to mock and between each other.

A key finding of our transcriptomic analysis was the significant downregulation of genes involved in oxidative phosphorylation and the electron transport chain in PAMs infected with NC field strains. In contrast, PIMs exhibited a more limited transcriptional response to infection with these strains. These gene expression changes in PAMs were consistent with the functional data obtained using Seahorse technology, which revealed a significant impairment of mitochondrial function, including reduced basal and maximal respiration, and spare respiratory capacity in PAMs infected with all tested PRRSV-2 strains. Notably, the NC strains, particularly NC134 and NC174, also significantly decreased coupling efficiency and cell respiratory control ratio in PAMs. The observed downregulation of oxidative phosphorylation-related genes in PAMs aligns with previous studies suggesting that PRRSV infection can induce mitochondrial dysfunction (26–28). This cellular reprogramming in PAMs is a key strategy that likely contributes to the impaired immune responses associated with PRRSV pathogenesis (58, 59). By crippling the oxidative phosphorylation impairment, PRRSV may further compromise the mitochondria-anchored antiviral signaling pathways mediated by mitochondrial antiviral signaling (MAVS) and retinoic acid-inducible gene I (RIG-I)-like receptor signaling, disabling one of the cellular alarm systems against viruses (60, 61). Our findings are consistent with a pathogenic strategy employed by other respiratory viruses that target AM metabolism. For example, the influenza A virus is known to induce immunoparalysis and decrease the enrichment of mitochondrial oxidative phosphorylation-related genes in AM (62). Similarly, transcriptomic signatures from AM of patients with SARS-CoV-2 revealed a low mitochondrial respiratory capacity (63).

The stronger transcriptional response in PRRSV-infected PAMs compared to PIMs is intriguing. PAMs, as the primary resident macrophage population in the alveoli and the first line of defense against airborne pathogens like PRRSV, appear to undergo a more pronounced metabolic shift upon infection compared to PIMs, which reside within the vascular endothelium and mainly fight blood-borne pathogens. This suggests that the virus may exert a more significant metabolic impact on PAMs during the early stages of infection, or there is a distinct interaction dynamic between the virus and the two types of macrophages.

The functional assays using Seahorse technology provided crucial validation of the transcriptomic findings. The consistent reduction in mitochondrial respiration across all tested PRRSV-2 strains in PAMs highlights the vulnerability of these cells’ bioenergetic machinery to viral interference. Specifically, the decreased spare respiratory capacity indicates a reduced ability of infected PAMs to meet increased energy demands during an immune response, potentially contributing to their functional impairment. The differences observed in coupling efficiency and respiratory control ratio, particularly with the NC strains, suggest a specific disruption in the efficiency of ATP production linked to oxygen consumption in PAMs infected with these isolates. The distinct metabolic profiles observed between PAMs and PIMs further emphasize the functional heterogeneity of these two macrophage populations in the context of PRRSV-2 infection. While ATP-linked respiration was impaired in PIMs by all strains, other metabolic parameters were less consistently affected, suggesting a different and less severe impact of PRRSV-2 on the mitochondrial function of PIMs at this early time point.

Interestingly, despite the mitochondrial dysfunction observed in infected macrophages, we did not detect significant differences in the production of ROS or NO between infected and control cells at the tested time points and MOIs. This finding contrasts with some previous studies that have reported increased ROS production during PRRSV infection (28, 64–66). However, the lack of NO production is supported by a previous PRRSV study using a similar approach and testing different MOIs (67). However, we used a different colorimetric approach and primary cells compared with previous studies, often evaluating the production of oxygen radicals during PRRSV-2 infection in cell lines (e.g., MARC145 or 3D4/2). The absence of significant differences in ROS and NO levels in primary macrophages could be attributed to several factors. First, the sensitivity of the detection systems used for ROS and NO may not have been sufficient to capture subtle yet significant changes in these reactive molecules. It is possible that production was present, but below the detection threshold of the assays employed. Second, mitochondrial dysfunction, particularly at early stages, may primarily manifest as impaired energy production rather than a substantial increase in ROS or NO. Cellular function may prioritize maintaining homeostasis by limiting the release of free radicals, especially when its overall energetic efficiency is compromised. This suggests a shift in metabolic priorities where the cell attempts to mitigate widespread damage. Finally, the altered organelle itself could prevent the release of free radicals. If dysfunctional mitochondria are unable to properly generate ROS and NO, then their accumulation within the organelle or their rapid neutralization by internal antioxidant systems could lead to undetectable extracellular levels. Additionally, we need to consider the differences between PRRSV strains and MOIs used in the different studies.

The validation of key transcriptomic changes using NanoString and RT-qPCR provided strong support for the RNA-seq data. The increase in IFNB and IL1B mRNA levels in PAMs and IFN-α protein in BAL MNPs upon infection, without significant differences between the NC strains, further confirms their similar pathogenetic mechanism.

The subtle transcriptional response we observed in PIMs may also stem from our experimental design. Our protocol involved infecting a heterogeneous culture of enriched parenchymal MNPs—which include both macrophages and dendritic cells—before sorting the target PIM population for RNA sequencing. This approach contrasts with the infection using bronchoalveolar lavage cells, which yielded a relatively pure population of PAMs. The initial heterogeneity of the parenchymal MNP culture could have diluted the PIM-specific response, potentially by reducing their per-cell infection efficiency or by modulating their activation through cell-to-cell interactions. This factor likely contributed to the high cross-replicate variability seen in the PIM gene expression data. Such variability inherently reduces the statistical detection of significant changes, explaining why significantly fewer DEGs were identified in PIMs compared to PAMs following PRRSV infection. The divergent PIM and PAM responses seem not related to differences in viral permissiveness because our analysis confirmed that viral loads were comparable between PAMs and PIMs for both the NC134 and NC174 strains of PRRSV. This finding strongly suggests that the differences in transcriptional signatures reflect intrinsic biological differences between these two macrophage populations rather than being related to viral replication levels. This conclusion is well-supported by previous studies demonstrating that PRRSV replicates to comparable levels in both macrophages *in vitro* (6, 13–15). Importantly, this is also corroborated by *in vivo* data showing similar viral read alignment in PAMs and PIMs sorted from pigs infected with the FL13 PRRSV-1 strain (12). Further dissecting the innate immune response, we found that while IFNA expression was induced similarly in sorted PAMs and PIMs post-infection, the overall IFN-α production from the bulk parenchymal MNP culture was significantly blunted compared to that of the BAL culture. This disparity likely arises from a combination of factors, including distinct response kinetics among the different cell types within the parenchyma and a generally weaker transcriptional activation, probably resulting from the heterogeneous nature of our infection model.

Our study has several strengths, including the use of primary porcine macrophages, the comparison of multiple NC field strains with a reference strain, the integration of transcriptomic and functional metabolic assays, and the implementation of Seahorse technology in the context of PRRSV and porcine lung macrophage research. However, we acknowledge some limitations. Our main experimental settings focused on the early stages of infection (12–16 h post-infection), and the long-term consequences of PRRSV-induced mitochondrial dysfunction remain to be elucidated. Additionally, the inherent high variability of primary macrophages and biological replicate responses may have reduced significant differences in our experimental layouts. Furthermore, while we examined two NC field strains in detail at the transcriptomic level, functional assays included an additional NC strain (NC144), providing a broader but not fully matched comparison. Future studies could investigate the temporal dynamics of mitochondrial dysfunction and oxidative stress over a more extended time course and at different MOIs and explore the underlying viral and host factors that contribute to strain-specific and cell type-specific differences in metabolic reprogramming.

## 5 Summary and conclusions

Our findings demonstrate that PRRSV-2 infection, particularly with North Carolina field strains, induces significant mitochondrial dysfunction in PAMs, characterized by a downregulation of oxidative phosphorylation pathways and impaired respiratory capacity. PIMs exhibited a less pronounced metabolic response at this early stage of infection. Interestingly, this mitochondrial impairment did not correlate with increased ROS or NO production at the measured time points. Seahorse technology was deployed for the first time in PRRSV macrophage research. Our results provide further insights into the early pathogenetic mechanisms of PRRSV-2 in key target cells and highlight the responses of PAMs and PIMs to infection with distinct North Carolina viral strains, contributing to a better understanding of the complex host–pathogen interactions that govern PRRSV pathogenesis.

## Data Availability

The datasets presented in this study can be found in online repositories. The names of the repository/repositories and accession number(s) can be found below: https://www.ncbi.nlm.nih.gov/geo/, GSE304527.
