# Supplementary material for: Mitochondrial dysfunction in PRRSV-2-infected macrophages
Source: Front Immunol. 2025 Oct 15;16:1670488. doi: 10.3389/fimmu.2025.1670488 (PMC12568354; doi:10.3389/fimmu.2025.1670488)
Supplement: Supplementary file 1 [file DataSheet1.docx]

Supplementary Material

# Supplementary Data

# Supplementary Figures and Tables

## Supplementary Figures


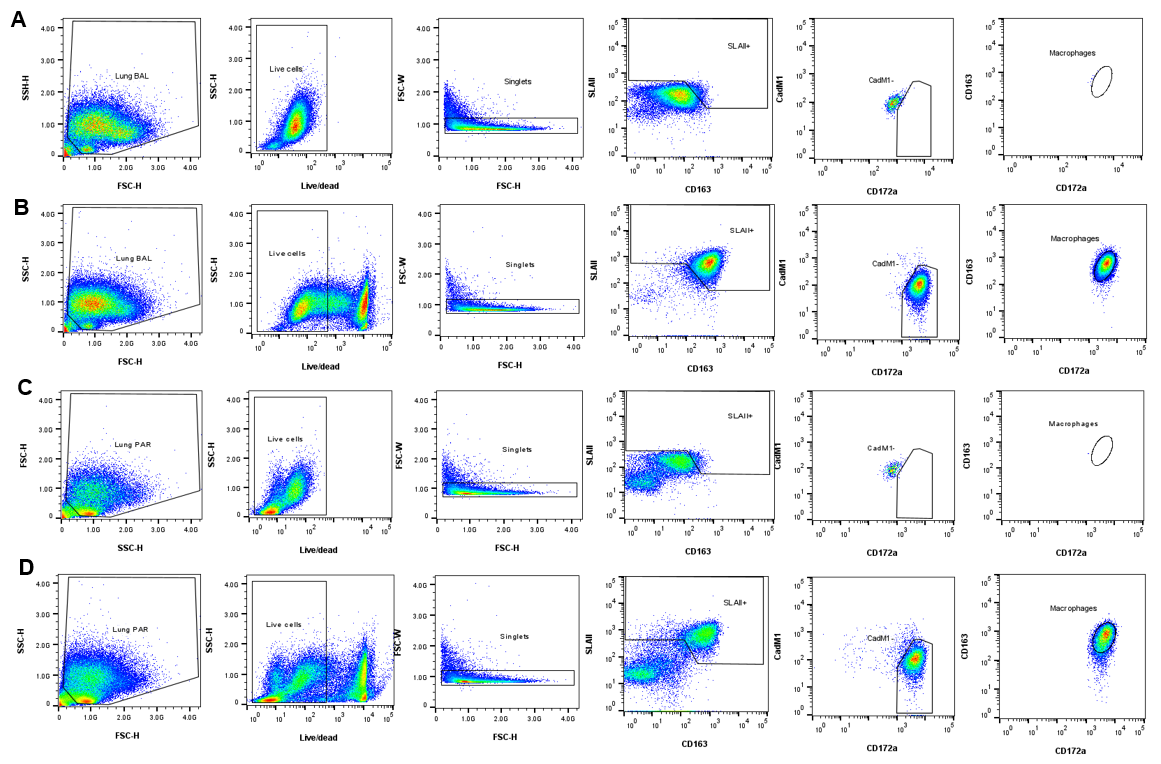


**Figure S1: Sorting strategy for PAM and PIM.** PAM (**A-B**), and PIM (**C-D**) were sorted from infected or control MNP at 12h post infection. A and C) Unstained control. Subpopulations were defined by using SLA-II/CD172a/CaDM1/CD163 markers. Each graph is the offspring of the previous gate from left to right. Data are representative of 4 independent sorting experiments.


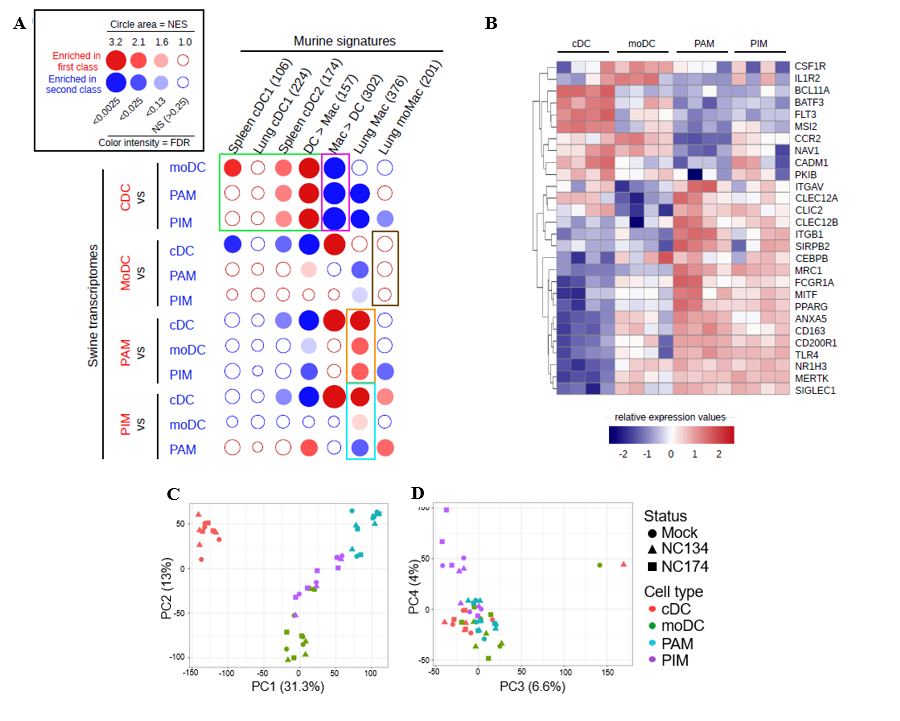


**Figure S2. Validation of the sorting strategy of swine MNP subsets. A**) Analysis of the homologies between swine and mouse MNP subsets by high-throughput GSEA using BubbleGUM. Gene signatures specific to each murine MNP were extracted following a methodology explained in (34). The murine signatures were assessed for enrichment in all possible pairwise comparisons between swine MNP subsets using the BubbleMap module of BubbleGUM. Results are displayed as Bubbles, bigger and darker for stronger and more significant enrichment, in a color matching that of the condition in which the signature was enriched. The strength of the enrichment is quantified by the Normalized Enrichment Score (NES) which represents the number and differential expression intensity of the genes enriched. The significance of the enrichment is measured by the false discovery rate (FDR) value (or q-value) representing the likelihood that the enrichment of the GeneSet was a false-positive finding. This q-value was further corrected for multiple testing, leading to a higher stringency of the significance threshold used. Gene numbers per signature are written in parenthesis. **B**) Heatmap showing a selection of key genes specific of swine MNP subsets, using the Euclidean distance and the Complete clustering method. N=4. **C-D**) Principal Component Analysis (PCA) of the expression profiles of macrophages, cDC and moDC. Left PCA plot showing PC1 vs PC2 and right PC3 vs PC4. PC contribution to the total variance is shown in parenthesis. N=4.


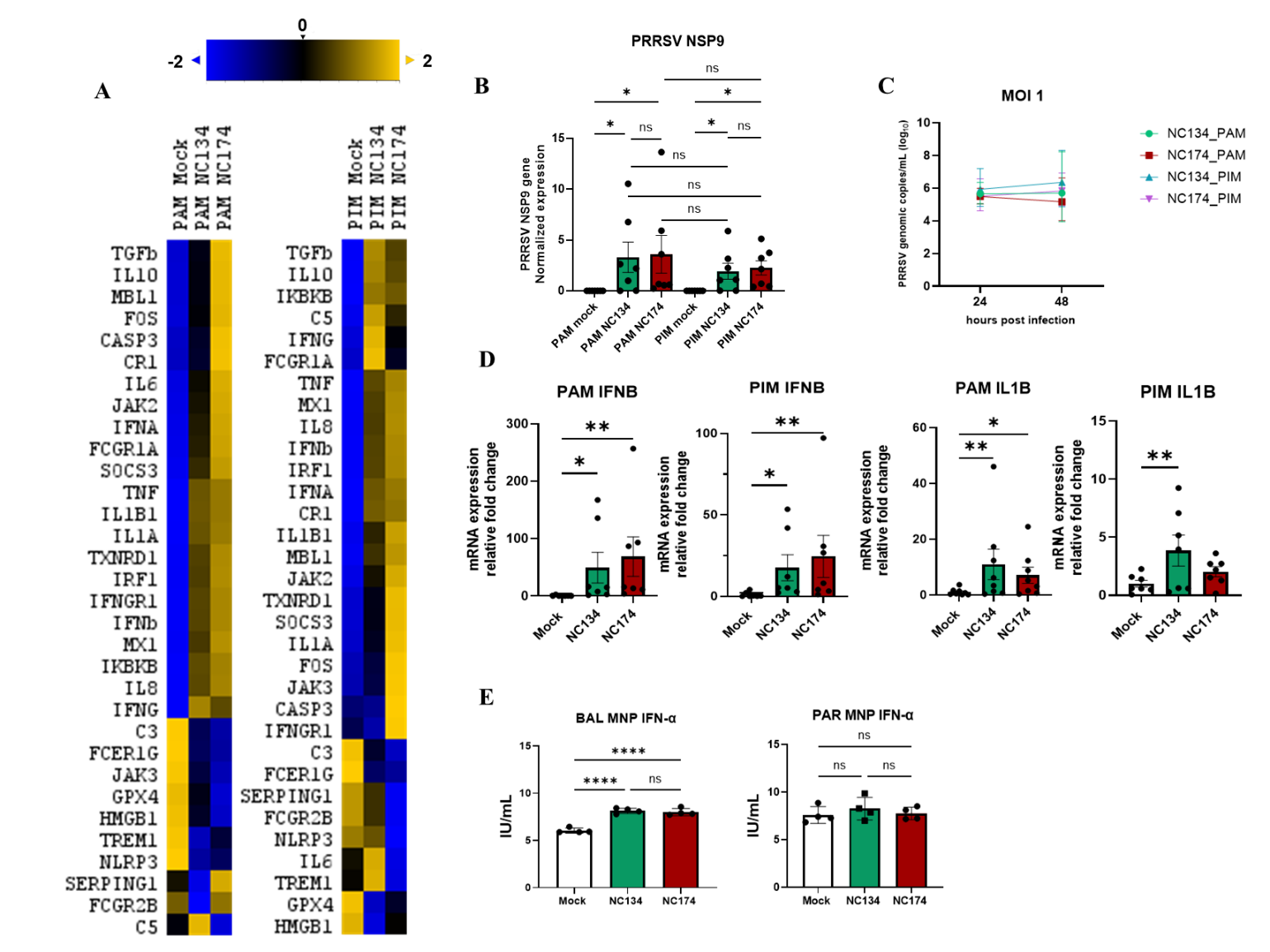


**Figure S3: NanoString, RT-qPCR, and IFN-α validation analysis. A**) NanoString heatmap of PRRSV2-infected PAM and PIM compared to controls. NanoString experiments were performed on additional samples of sorted cells infected at MOI 1 for 12h. N=4. For the analysis a custom CodeSet of 46 genes was used (Supplementary Table 1). RNA counts were analyzed and exported using nSolver analysis software (nSolver 4.0 Analysis Software, Inc., Seattle, WA, USA). Background levels were calculated as geometric means of negative controls ± 2* standard deviation (SD) and expression data below the background were considered negative. **B-D**) Additional sorted cells and cDNA remaining from libraries preparation were used for RT-qPCR. To reduce the variation between samples in the RT-qPCR raw data, the Ct values of the housekeeping gene were used to perform a ΔCt normalization. Subsequent normalization to the mean of the mock values was performed for IL1β and IFNβ expressions (**D**). **C**) NC134 and NC174 virus kinetic growth in PAM and PIM at 24h and 48h post infection. N=4. **E**) Supernatants from infected PAM and PIM and control cultures used for the sorting were examined for IFN-α by capture ELISA at 12h post infection. Data are shown in IU/ml. Statistics: Ordinary one-way ANOVA. * p < 0.05 ** p < 0.01. *** p < 0.001 **** p < 0.0001. ns = not significant.

**
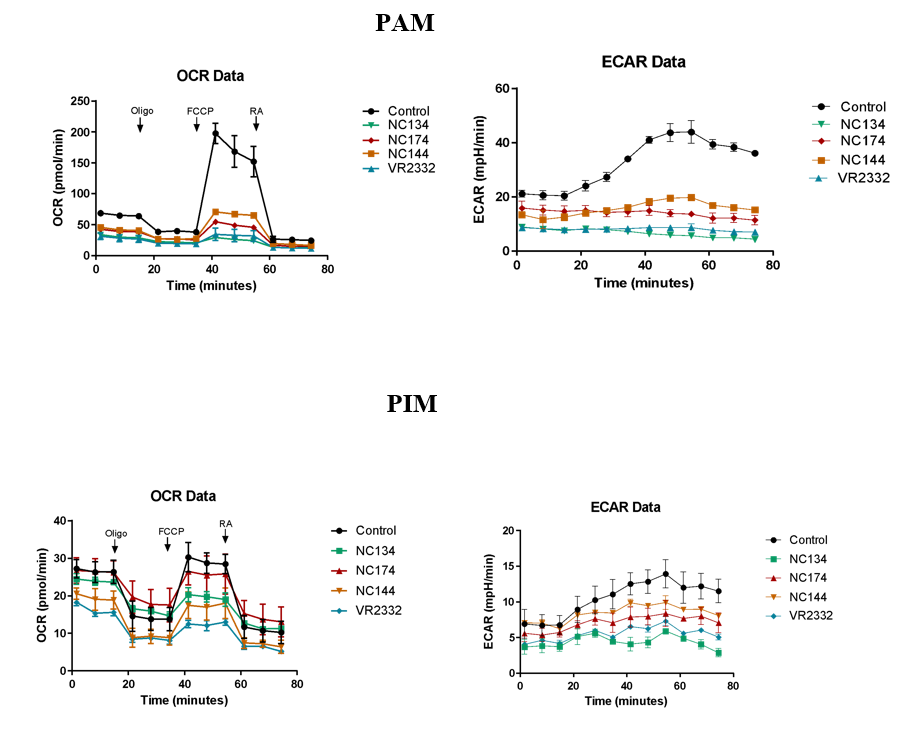
**

**Figure S4: Mitochondrial dysfunction in fresh PAM and PIM infected with PRRSV-2.** Figures correspond to representative figures of the key components of mitochondrial respiration in the Seahorse Cell Mito Stress assay, including Oxygen Consumption Rate (OCR) profiles and Extracellular Acidification Rate (ECAR) in infected and control PAM. Oligomycin (Oligo) reduces the oxygen consumption ration (OCR) through inhibition of ATP synthase, a critical facilitator of oxidation phosphorylation of ADP into ATP. Carbonyl cyanide-4 (trifluoromethoxy) phenylhydrazone (FCCP) potently collapses the proton gradient at the inner mitochondrial membrane, simulating a maximal energy demand in the cells. Rotenone and antimycin A are complex I and complex III inhibitors of the electron transport chain (ETC). Data was analyzed in Wave software (Agilent) and visualized using GraphPad Prism v10.

## Supplementary Tables

**Table S1.** NanoString custom CodeSet of 46 pig genes.

| **Gene** | **Accession No.** | **Class Name** |
| --- | --- | --- |
| *C3* | NM_214009.1 | Endogenous |
| *C5* | NM_001001646.1 | Endogenous |
| *CASP3* | XM_005671704.2 | Endogenous |
| *CD83* | XM_001928655.4 | Endogenous |
| *CD86* | NM_214222.1 | Endogenous |
| *CR1* | XM_013979663.1 | Endogenous |
| *FCER1G* | NM_001001265.1 | Endogenous |
| *FCGR1A* | NM_001033011.1 | Endogenous |
| *FCGR2B* | NM_001033013.2 | Endogenous |
| *FOS* | NM_001123113.1 | Endogenous |
| *GPX4* | NM_214407.1 | Endogenous |
| *HMGB1* | NM_001004034.1 | Endogenous |
| *IFNA* | NM_214393.1 | Endogenous |
| *IFNG* | NM_213948.1 | Endogenous |
| *IFNGR1* | NM_001177907.1 | Endogenous |
| *IFNB* | NM_001003923.1 | Endogenous |
| *IKBKB* | NM_001099935.1 | Endogenous |
| *IL10* | NM_214041.1 | Endogenous |
| *IL12P35* | NM_214013.1 | Endogenous |
| *IL12P40/IL23* | NM_213993.1 | Endogenous |
| *IL17* | NM_001005729.1 | Endogenous |
| *IL1A* | NM_214029.1 | Endogenous |
| *IL1B1* | NM_214055.1 | Endogenous |
| *IL6* | NM_214399.1 | Endogenous |
| *IL8* | NM_213867.1 | Endogenous |
| *IRF1* | NM_001097413.1 | Endogenous |
| *JAK2* | NM_214113.1 | Endogenous |
| *JAK3* | XM_003123500.3 | Endogenous |
| *MBL1* | NM_001007194.3 | Endogenous |
| *MX1* | NM_214061.1 | Endogenous |
| *NLRP3* | NM_001256770.1 | Endogenous |
| *SERPING1* | NM_001123194.1 | Endogenous |
| *SOCS3* | NM_001123196.1 | Endogenous |
| *TGFB* | NM_214015.1 | Endogenous |
| *TNF* | NM_214022.1 | Endogenous |
| *TREM1* | NM_213756.1 | Endogenous |
| *TXNRD1* | NM_214154.3 | Endogenous |
| *GAPDH* | NM_001206359.1 | Housekeeping |
| *HMBS* | NM_001097412.1 | Housekeeping |
| *HPRT1* | NM_001032376.2 | Housekeeping |
| *RPL32* | NM_001001636.1 | Housekeeping |
| *RPL4* | XM_005659862.2 | Housekeeping |
| *RSP24* | XM_001929385.6 | Housekeeping |
| *SDHA* | XM_013992340.1 | Housekeeping |
| *TBP* | XM_013991786.1 | Housekeeping |
| *TOP2B* | NM_001258386.1 | Housekeeping |

**Supplementary table 2**: DEGs for PAM NC134, PAM NC174, PIM NC134, PIM NC134vs NC174

**Supplementary table 3**: enrichR pathways for PAM NC134 updown GO BiolProc, PAM NC134 updown MSigDB Hall, PAM NC174 updown GO Biol Proc, PAM NC174 updown MSigDB Hall, PIM NC134 updown GO BiolProc, PIM NC134 updown MSigDB Hall
